# A Lecture on Aortic Disease

**Published:** 1909-12-04

**Authors:** Frederick Taylor

**Affiliations:** Physician to the Seamen's Hospital, and Consulting Physician to Guy's Hospital


### Hospital Clinics.

### A LECTURE ON AORTIC DISEASE.

By FREDERICK TAYLOR, M.D., F.R.C.P.; Physician to the Seamen's Hospital, and Consulting Physician to Guy's Hospital.

(Delivered at the Seamen's Hospital, Greenwich, on Wednesday, November 3, 1909.)

Gentlemen,—I have chosen for to-day's lecture the subject of aortic disease, because it is one with which we are all, more or less, in constant contact; and though no doubt nearly everything has been said about it that can be said, still, as in nearly all subjects, there hang around the admitted facts a large number of debateable points possessing interest, and sometimes considerable importance. Aortic disease, which is a comprehensive term, may be held to include aneurysm, valvular disease, and growths in connection with the aorta. I propose to deal with it chiefly from the point of view of cases in which there is evidence of disease at the base of the heart, whether in the form of valvular disease or of dilatation of the aorta above the valve. And I propose to talk of the matter chiefly from the clinical side. I may remind you that disease of the valves and orifice is mainly dependent on two conditions: (1) acute endocarditis, such as is found especially in connection with rheumatic fever and some other forms of infectious diseases; and (2) arterio-sclerosis, the condition which formerly used to be known by the name of atheroma, and which we know of mainly as a degenerative, and possibly in some degree inflammatory, change occurring in later life. And we may still further premise that the pathological changes which take place as the result of endocarditis or of arterio-sclerosis are those which destroy the efficiency of the valve as a valve; that is, they may narrow the orifice or it may interfere with the position and structure of the valves so as to prevent them closing efficiently. The obstructions that we find as the result of disease may be masses of vegetation upon one or other valve, or there may be union of the valves together, so that they do not lie back against the wall of the aorta. As causes of regurgitation, we have the possibility of the valves being shrunken in structure, which prevents the closure of the orifice, when the recoil of blood takes place; or there may be one valve shrunken, or retroverted, so that its margin does not come into contact with those of the other valves. Or there may be atheromatous changes at the root of the aorta, leading to such a dilatation that the valves are unable to meet.

### Murmurs in Aortic Cases.

Turning to the clinical side of the question, we are all familiar with the fact that murmurs occur in connection with these changes. If either of the two physiological sounds of the heart becomes imperfect and lengthened into a murmur, that is an indication of some imperfection in the valvular apparatus. In the case of a systolic murmur, the blood is being driven from the ventricle into the aorta; the murmur then is due to the jet which passes through from a wide orifice into a narrow space beyond. The murmur is sometimes attributed to the vibration of the edges of the valve. That may be true in part, but there is no doubt that vibrations take place in the fluid, apart from the valves. Even when the edges of the valves are rigid there is a murmur, and that vibration in the fluid is admitted to be the main cause of the sounds which we find in these circumstances. When there is a systolic murmur like that, in a certain number of cases it is due to an obstruction of the aortic orifice by something wrong with the valves: either union of the valves, or growths, or vegetations on them, or some thickening which prevents the valve lying properly back, and allows it to be in the way of a main stream. But as this is not the only condition which may cause a systolic murmur, you are not hastily to diagnose obstructive disease of the aorta because you hear a systolic murmur which is traceable upwards towards the clavicle and occurs in systole. The orifice may be normal and the valve soft, and blood may pass easily into the aortic orifice; yet if the aorta is dilated beyond that, there are the conditions for the production of a murmur. Since this condition arises in cases of arterio-sclerotic disease, a systolic murmur in older people does not necessarily mean that they have valvular disease, for they may have only aortic dilatation beyond the valves. If that is the case, your diagnosis must depend on other conditions, and one of those is the likelihood of either endocarditis, or of arterio-sclerotic disease being present. This you can generally easily decide. If it occurs in a young person, æt. 25 or even 30, it is more likely to be the result of endocarditis due to rheumatism, of which the patient may or may not be cognisant. But if the patient is æt. 40 or more, and there is no history of rheumatism, it is probably arterio-sclerotic.

### The Diastolic Murmur.

Now, passing to the diastolic murmur, there are interesting points as to the position in which it is heard. It is sometimes heard at the second right costal cartilage, is traceable a little way down, and disappears. There are cases in which it is not heard at that point, but yet is heard lower down, along the sternum or on one or other side of it; and if you are listening for aortic regurgitant murmurs you must not confine your attention to where you think the base of the aorta is. There are cases of double murmurs also in which that part of the chest is almost entirely free from sound; but working upwards you hear a systolic murmur, and downwards a diastolic. It has been thought possible that the different positions of these murmurs can be explained by lesions of different valves. The suggestion is that, whichever of the valves is diseased, the escape of blood will be on that side of the aorta, and so the position of the murmur would be a guide to the valve concerned. But I have always had a difficulty in believing it, because, as far as we know, the leakage must be somewhere near the centre of the aorta, and whichever valve is defective I take it the escape would be nearly at the centre of the current. Of course if there were a hole in the centre of one or other valve I can understand some difference might take place. Now we know two of these valves are related to the openings of the coronary arteries, and the suggestion has been made that in the event of disease being recognised as belonging to one of the valves in connection with a coronary artery, one might, from the possibility of this coronary orifice being involved in the disease of the valve, give a worse prognosis than one would if there were a lesion of the valve in which no coronary artery was concerned. I doubt if this has been found to be of great practical value.

### Pulmonary Regurgitation Murmurs.

Is a diastolic murmur along the left side of the sternum necessarily aortic? Certainly not. We have to recognise that there are cases in which there is regurgitant disease of the pulmonary valves; and though pulmonary regurgitant murmurs are not common, yet they do occur. Some years ago they were regarded as very exceptional. When, twenty-five years ago, a case was brought before the Clinical Society it was considered such a rarity that a special committee of the Society was appointed to investigate and report upon it. But since that time the possibility of such murmurs occurring under these circumstances has been recognised. In mitral stenosis the obstruction to the blood at the mitral orifice leads to enormous tension in the left auricle, and, pressing backwards, that tension has its effect on the pulmonary circulation, the pulmonary artery gets distended, and then occasionally it appears that regurgitation has taken place through the pulmonary valves. That being the case, of course the murmur has been heard in association with the evidences of mitral obstructive disease; and the murmur has generally been heard down the left side of the sternum, in a position where, it is true, some aortic murmurs can be heard. So, that if there is very obvious pulmonary stenosis and the patient has had it for a long time, if there is no evidence in the pulse of the existence of aortic regurgitation, and if the pulse is like one of mitral stenosis and not like one of aortic regurgitation, you have to consider the possibility of its being an instance of pulmonary regurgitation. There are two other circumstances in which you get a pulmonary murmur. Infective endocarditis may cause acute disease of valves leading to obstruction and regurgitation. It may also occur in congenital disease involving the pulmonary artery, and there may be occasionally regurgitation as well as obstruction.

Another question in regard to aortic regurgitant murmurs is this: Does it necessarily follow that the patient should have all the evidence of aortic disease because there is an aortic murmur? May not the aortic murmur be the very first sign of aortic disease? A characteristic result of aortic disease is the pulse which has been described as the regurgitant pulse, the splashing pulse, the water-hammer pulse, or Corrigan's pulse; and which is thought to be as diagnostic as anything in the history of the case. It is important to consider whether directly a patient gets aortic disease he should have these effects produced on the pulse. It seems to me that he need not. The first effect of leakage through a valve is that a small quantity of blood regurgitates, and it is easy for that small quantity, being forced through that orifice, to acquire the vibrations which cause the noise. And yet that quantity of blood may be insufficient to have any effect on the pulse such as we see in more advanced cases.

Another point of some interest is the occurrence of the so-called Flint's murmur. The fact was recognised some years ago, and enlarged upon by Dr. Austin Flint, that in some cases of aortic regurgitation a murmur is heard at the apex of the heart which is like the murmur of mitral stenosis: there is an audible diastolic aortic murmur, and perhaps other indications of completely developed aortic disease, and the patient at the same time has a murmur like that of mitral stenosis—a presystolic rumbling murmur, sometimes with a thrill, sometimes with a loud first sound—so that mitral stenosis is, in some of these cases, diagnosed, and yet, when the cases are examined post-mortem, there is no mitral disease. For myself, I have not regarded Flint murmurs as very common, but in the last few years statements have been made that Flint's murmur occurs in something like half the cases of aortic regurgitation. And if that is the case, it is of great importance, because you may wrongly diagnose the existence of stenosis when it is present, and when the physical signs are really due to the regurgitation through the aortic valve.

You may ask what is the explanation of the Flint murmur? There are two or three views with regard to that. The first suggestion is that the regurgitating current of blood through the ventricle floats up the mitral valves so that they oppose the blood coming in from the auricle. Blood comes in from the aorta through the leakage, and blood is flowing in from the auricle through the mitral valve; the regurgitating current floats up the valves against the auricular current, and causes an obstruction, so that the same kind of murmur is produced as if that obstruction were a permanent result of the thickening of the mitral cusps. Another view is this: That the aortic current plays against the anterior mitral cusp, and perhaps the auricular current is playing against it at the same time, and those two currents, impinging upon the mitral curtain, make a sound. The interesting question will be to ask why they are produced in only half the cases.

We are familiar with the fact that aortic disease, like other valvular lesions, leads to hypertrophy and dilatation. In this there are the greatest degrees of dilatation which you meet with anywhere; bovine hearts, which weigh 48 ozs., are generally the result of aortic disease. In that case the heart enlarges outwards and downwards, especially downwards. I have already pointed out that aneurysm and aortic disease are often associated together, and it may be difficult to estimate how much of the disability is due to the one and how much to the other. I wish to remind you that if there is a hypertrophied heart in a case in which you have reason to suspect aneurysm or in which you know aneurysm exists; for instance, pulsation in the right intercostal spaces of considerable degree, or pain in that region, with a basal murmur or murmurs; and if the heart is displaced outwards and downwards, you have to consider whether that is due to hypertrophy or not. It is well attested that if there is hypertrophy and dilatation of the left ventricles, the valves must be affected. I do not say necessarily diseased. An aneurysm of itself above the valves untouched functionally and structurally does not cause hypertrophy of the heart. That does not mean that every time you feel a heart displaced from the normal position there must be aortic valve disarrangement, because a large aneurysm might push the heart down. Aneurysm may spread to the valvular ring, and so lead to regurgitation. So that, if there is hypertrophy you have to remember that the valvular apparatus is probably diseased, and that is important in relation to your attempts at treatment, as well as in relation to prognosis; because if the valvular apparatus is out of order in addition to aneurysm, the cure of the aneurysm, even if it can be attained, is less likely to be productive of long life, and the prognosis is much worse.

Patients with aortic disease get breathless, and in the later stages anæmia is a very striking feature. But in the later stages we know the mitral valve becomes involved, and regurgitation takes place. The patient gradually reaches that condition which we are familiar with in mitral disease, and we observe engorgement of the lungs, œdema, dropsy, enlargement of the liver, pulsation of the liver, perhaps, with enlargement of the spleen, ascites, and ultimately a fatal result.

The question of treatment now comes in, which I have only time to touch on briefly. What can be done in definite aortic disease? Little can be done for the valves themselves. We do not know much about the means for improving valves. The great question in all cardiac affections is the maintenance of so-called compensation; that is to say, when disease of valves takes place, so that they do not work as they ought, the strain is thrown on the muscular apparatus of the heart. That apparatus may at first suffice for all that is wanted. As long as it does so, the patient does not suffer, and compensation is said to be maintained. But when the heart has more work to do in consequence of a valvular lesion, the ventricle or auricle dilates, inefficient contractions occur, and the compensation is said to have broken down. You advise in these cases rest, so as to diminish the strain upon the circulation; and you can also frequently further this object by giving purgatives, diuretics, and diaphoretics. In relation to aortic disease the interesting question has been raised whether or not digitalis should be given. I think there is no doubt, in the universal opinion of those who are authorised to speak on the question, that digitalis may certainly be used tentatively in any case in which dilatation of the heart has proceeded to a point that renders it ineffective. Digitalis is not wanted in numbers of cases of aortic disease in which the heart is hypertrophied and is beating quietly and steadily, though perhaps not with a perfect result. The point is not so much that you will do harm by giving it, but that you will not do good. The action of digitalis is almost restricted to the cases in which the heart is dilated, and the beats of the heart are rapid and irregular and ineffective. In those cases you are not to be afraid of using digitalis simply because your case is labelled aortic disease. One of the reasons given for withholding it is that it lengthens the diastole, and it is conceived that if you lengthen the diastole of a heart in which the blood is regurgitating through the aortic valve, the cerebral circulation will be depleted and the patient will have syncope, or his vital functions may suddenly cease. I have frequently enough given digitalis in aortic disease, and I think it is the universal practice to use it in carefully selected cases and with sufficient precautions. But generally in aortic disease the treatment must depend very much on adequate rest, upon suitable purgatives, and upon moderate, but of course sufficient, diet. The patient with aortic disease frequently suffers from throbbing of the vessels of the neck, from the extreme excursions which the vessels may make. It is a difficult symptom to deal with. I have tried bromide of potassium, which seems a remedy likely to be useful.

**The Administration of Santonin.**

In view of the greater toxicity of santonin to the human being than to the ascarides, Dr. Pélissier insists on the necessity of administering the drug under special conditions and in such a vehicle that the poison may reach the parasite without being absorbed by the patient. The preparatory treatment consists in administering at bedtime and the following morning, fasting, the following mixture: One clove of garlic, cut small, and one glass of milk: cook for ten minutes over a slow fire, strain, and sweeten to taste. The cooking deprives the garlic of its acrid properties and renders the preparation agreeable to take. The object of this drink is to render the parasites more vulnerable by modifying their conditions of existence, and to introduce into the stomach an essential oil which excites the acid secretion and diminishes the capacity for absorption of the medicament. Santonin should be given some minutes after the above mixture in dose according to age (generally 0.01 centigramme for each year of age up to 0.30 centigramme), with almond oil 5 gms, and syrup of gum 30 gms. in orange-flower water 60 mils. This is taken in three draughts, with five-minute intervals. In the morning lemonade is given. Two hours after taking the santonin purgation by calomel. Santonin thus dissolved in oil passes through the stomach without being touched by the digestive fluids; the dose, taken directly, reaches the parasite, upon which all its toxicity is exerted.

